# Dynamics and correlations of chlorophyll and phytol content with silage bacterial of different growth heights *Pennisetum sinese*

**DOI:** 10.3389/fpls.2022.996970

**Published:** 2022-10-13

**Authors:** Mao Li, Renlong Lv, Hanlin Zhou, Xuejuan Zi

**Affiliations:** ^1^ Tropical Crops Genetic Resources Institute, Chinese Academy of Tropical Agricultural Sciences, Danzhou, China; ^2^ Zhanjiang Experimental Station, Chinese Academy of Tropical Agricultural Sciences, Zhanjiang, China; ^3^ Key Laboratory of Ministry of Education for Genetics and Germplasm Innovation of Tropical Special Trees and Ornamental Plants, Key Laboratory of Germplasm Resources of Tropical Special Ornamental Plants of Hainan Province, College of Forestry, Hainan University, Danzhou, China

**Keywords:** *Pennisetum sinese*, growth height, chlorophyll, phytol, silage microbial

## Abstract

The dynamics and correlations of chlorophyll and phytol content with silage bacterial of different growth heights *Pennisetum sinese* were investigated. The results demonstrated that the chlorophyll and phytol content of *P. sinese* before and after ensiled decreased with the increase of growth height. Ensiling significantly reduced pigment content but had no significant effect on phytol. In addition, *P. sinese* pigment yield before and after ensiled increased with growth heights increasing, and the yield at 150 or 180 cm was obviously higher. Moreover, the higher silage quality V-Score were at 150 or 180 cm growth heights. Furthermore, the silage microbial diversity were varied by growth heights, and some specific undesirable microorganisms (*Acinetobacter*, *Cellvibrio*, *Sphingobacterium*, etc.) were negatively correlated with pigment and phytol content. Therefore, with comprehensive consideration of pigment, phytol yield, and silage quality, the optimum harvest growth height of *P. sinese* was 150 cm. Furthermore, precise reduction of particular undesirable microorganisms maybe helps to preserve pigments and phytol.

## Introduction

In recent years, consumers are becoming more and more concerned about the healthy functions of animal products (Eggersdorfer and Wyss, 2018; Liu et al., 2020). Phytanic acid is an important functional component, which could regulate the oxidation of fatty acids, reduce the incidence of some types of cancer, and relieve fatty liver and diabetes (Wright et al., 2014; Kwan et al., 2015; Roca-Saavedra et al., 2017; Lv et al., 2020). The main sources of phytanic acid are milk and beef; hence, the concentration of phytanic acid is one of the most important indexes to evaluate functional animal products, especially for ruminants (Vetter and Schroder, 2010). In addition, phytol in chlorophyll is a synthetic precursor of phytanic acid, which directly determines phytanic acid concentration (Lv et al., 2017). Moreover, phytol has positive effects on fat metabolism, meat quality, muscle development, and regulation of animals, which further affects animal health and animal product quality (Yang et al., 2017; Bobe et al., 2020; Ding et al., 2021). In general, the content of functional components in animal products is mainly associated with feed and animal digestion and absorption (Liu et al., 2020). Therefore, phytol or chlorophyll content in forage will affect the accumulation of phytanic acid in animal products, and providing ruminants with higher level of phytol or chlorophyll diet will help to produce high-quality animal products (Santra et al., 2021).

According to previous reports, the contents of phytol or chlorophyll in forage were affected by many factors, for instance, forage variety and growth period (Lv et al., 2021). In addition, cultivation measures affect these pigment content of forage, Hák et al. (1993) and Lv et al. (2017) reported that the chlorophyll and phytol contents increased when applied nitrogen fertilizer and harvested earlier. Moreover, the forage processing and modulation method also affects the pigment content; Lv et al. (2021) reported that drying can obviously reduce the content of chlorophyll and phytol in the harvest grass. On the contrary, ensiling could preserve the phytol in temperate grasses Italian ryegrass well (Lv et al., 2017; Lv et al., 2020). Phytol is produced by chlorophyll degradation in acidic environments, and the acidity of silage may be the key factor of phytol conversion. During the ensiling, pH value was mainly affected by the silage microbial community (Du et al., 2022; Zi et al., 2022). Therefore, the resolving of silage microbial community structure may help to improve the efficiency of phytol conversion.

*Pennisetum sinese* is typical tropical grass and important ruminant feed, which also was C4 plant with high photosynthetic efficiency (Sanborn et al., 2021). Due to multiple cutting, *P. sinese* is hard to judge its nutritional value through its growth period. To some extent, the growth heights reflect the growth and development stages of plants, which influenced chemical composition and feed quality (Dong et al., 2013; Zi et al., 2017). Therefore, we speculate that *P. sinese* contains more photosynthetic pigments, and the impact of growth heights on the phytol or chlorophyll level also should be clarified. However, the contents of phytol or chlorophyll in *P. sinese* were still unknown. Meanwhile, the role of silage microorganisms in the preservation of phytol was also unclear. The purpose of this study was to clarify these doubts; thus, we investigated the dynamics and correlations of chlorophyll and phytol content with silage bacterial of different growth heights *P. sinese*.

## Materials and methods

### Location, grass production, and sample preparation

*Pennisetum sinese* (*P. purpureum × P. glaucum* cv. Reyan No.4) was planted at the experimental base of Chinese Academy of Tropical Agricultural Sciences (CATAS) in Danzhou, Hainan, China (longitude of 109°30′ E, latitude of 19°30′ N, altitude of 149 m). The average annual rainfall in this area is 1,815 mm with a mean annual temperature of 23.3°C. A completely randomized design was used in the present study, *Pennisetum sinese* of four plant height (approximately 90, 120, 150, and 180 cm) were harvested, each plant height was assigned into three randomly allocated plots with each plot measuring an area of 5 m × 5 m, and each plot was 0.5 m apart. *Pennisetum sinese* were planted on February 2018 and planting distance was 40 cm between rows, which was fertilized at a rate of 120-kg Urea, 150-kg P_2_O_2_, and 120-kg K_2_O ha^-1^, and watering was carried out 1 day each week except that when natural precipitation occurred during these days. Weed, pest, and disease control were conducted once a month. *Pennisetum sinese* was cut 3 cm above the ground on 28 February 2018 (plant height 90 cm, H1), 16 March 2018 (plant height 120 cm, H2), 29 March 2018 (plant height 150 cm, H3) and 23 April 2018 (plant height 180 cm, H4), respectively. Weighed the cut grass and calculated the yield of each height (three plots), and the harvested grass was chopped into 2 cm using a grass chopper (9Z-2.5, Zhengzhou Jinhongxing

Industrial Co., Ltd., Zhengzhou China). Then, the mixed well grass spread out in the shade about 4h and wilted to approximately 80% moisture content. Then, 100 g of above sample was stored at −80°C for analysis of pigment analysis and 100 g sample was dried at 65°C for 48h for chemical composition analysis, each height had three replicates (AOAC, 2000).

### Silage preparation

Above wilted *Pennisetum sinese* samples (200 g) was blended and vacuumed (Chunze Vacuum Sealer, Chunze Machinery Technology Co. Ltd, Weifang, China) in plastic bags (Synthetic resin, 30 cm × 10 cm × 4 cm; Henghou Packing Co. Ltd, Jiangmen, China). A total of 12 bags (four heights × three replicates) were prepared and incubated at room temperature (25°C–30°C). After 60 days ensiling, the bags were opened, 50 g of silage was stored at −80°C for analysis of pigment analysis and 100 g silage was dried at 65°C for 48h for chemical composition analysis (AOAC, 2000). In addition, 50 g silage was blended with 200 ml of distilled water, followed by incubation at 4°C for 24h and then filtration (Zi et al., 2022). Half of each extract sample was stored at −80°C for analysis of the microbiota diversity and the rest of extract sample stored at 4°C for 24h for fermentation quality analysis (Zi et al., 2022).

### Chemical analysis

Specimens were heated at 65°C for 72h, and dried materials were ground for chemical analysis. The contents of dry matter (DM), crude protein (CP), neutral detergent fiber (NDF), and acid detergent fiber (ADF) were measured as previously described (Van Soest et al., 1991; AOAC, 2000). CP values were calculated used nitrogen value multiplied by 6.25. Heat-stable amylase and sodium sulfite were used in the NDF procedure, and the results were expressed without residual ash. The pigments and phytol was determined as reported previously by Lv et al. (2017), the above frozen grass and silage samples were freeze-dried for 72h and extracted with 80% acetone, the chlorophyll a and chlorophyll b in the acetone extract were analyzed by HPLC, and the phytol was analyzed by gas chromatography (GC) after saponification and purification of the acetone extracts. The silage fermentation quality was determined using above extracts. The pH was measured with a glass electrode pH meter. The levels of lactic acid, acetic acid, propionic acid, butyric acid, and NH_3_ -N/total N were determined as previously established (Liu et al., 2012). V-Score was calculated from the NH_3_-N/total N and organic acid concentrations (Chen et al., 2016).

### Microbial community analysis

The extracts described above were used to analyze the microbial communities. Total DNA was isolated using the Stool DNA Kit (OMEGA Bio-Tek, Norcross, GA, USA). The V3–V4 regions of 16*S* rDNA were amplified using the thermocycler PCR system (GeneAmp 9700, ABI, Los Angeles, CA, USA) and primer sequence: 338F (5′- ACTCCTACGGGAGGCAGCAG-3′) and 806R (5′-GGACTACHVGGGTWTCTAAT-3′). Then, the high-quality PCR products sequencing was carried out using an Illumina MiSeq 2500 platform (Illumina, Inc., San Diego, CA, USA). The microbial diversity was assessed using the alpha diversity indices: Shannon and Simpson index. Beta diversity: principal components analysis (PCA) and Unweighted Pair-group Method with Arithmetic Mean (UPGMA) were conducted. The linear discriminant analysis (LDA) effect size (LEfSe) method was employed to identify the bacterial strains with different abundances among groups. The *P. sinese* silage microbial (genus) and pigments and phytol content were used R software to generate a heat map. Above methods details were as previously described (Zi et al., 2021; Zi et al., 2022). The data were analyzed using the free online BMKCloud Platform (www.biocloud.net). The sequencing data were deposited in the Sequence Read Archive (SRA) with the accession number of PRJNA637294.

### Statistics

The effects of plant heights were evaluated by one-way analysis of variance using the general linear model procedure of SAS 9.3 software (SAS Institute Inc., Cary, NC, USA). Differences were compared using Duncan’s multiple range test, and differences with *P* < 0.05 were considered statistically significant.

## Results

### Dynamics of chemical composition, chlorophyll, phytol, and biomass yield of *Pennisetum sinese* before ensiled

The dynamics of chemical composition, chlorophyll, phytol, and biomass yield of *Pennisetum sinese* at different plant heights are shown in **Table 1**. The contents of NDF and ADF significant increased (*P* < 0.01) with the increase of plant height, whereas the CP content and RFV decreased significantly (*P* < 0.01). Meanwhile, the plant height also affects pigment content. The biomass yield increased with plant height increasing (*P* < 0.01), but the chlorophyll and phytol contents decreased with plant height increasing (*P* < 0.01).

**Table 1:** The chemical composition, chlorophyll, phytol, and biomass yield of *Pennisetum sinese* at different heights.

	H1	H2	H3	H4	SEM	*P*-value
Crude protein (g/kg DM)	211^a^	192^b^	176^c^	130^d^	1.15	< 0.01
NDF (g/kg DM)	510^d^	547^c^	565^b^	607^a^	12.2	< 0.01
ADF (g/kg DM)	280^a^	315^b^	336^c^	352^c^	10.9	< 0.01
RFV	122.4^a^	109.5^b^	103.3^b^	94.2^c^	5.8	< 0.01
Chlorophyll a (g/kg DM)	6.35^a^	4.47^b^	3.68^c^	2.31^d^	0.46	< 0.01
Chlorophyll b (g/kg DM)	2.11^a^	1.31^b^	0.93^c^	0.72^d^	0.24	< 0.01
Chlorophyll (a+b) (g/kg DM)	8.46^a^	5.78^b^	4.62^c^	3.03^d^	0.38	< 0.01
Phytol (g/kg DM)	3.12^a^	1.90^b^	1.52^c^	1.18^d^	0.11	< 0.01
Biomass yield (t/ha DM)	6.2^d^	9.9^c^	13.6^b^	19.7^a^	0.16	< 0.01

SEM: standard error of means. Means within the same row with different letters are significantly different (P < 0.05).

### Comparation of the content and yield of chlorophyll and phytol of *Pennisetum sinese* at different heights before and after ensiling

**Figure 1:**
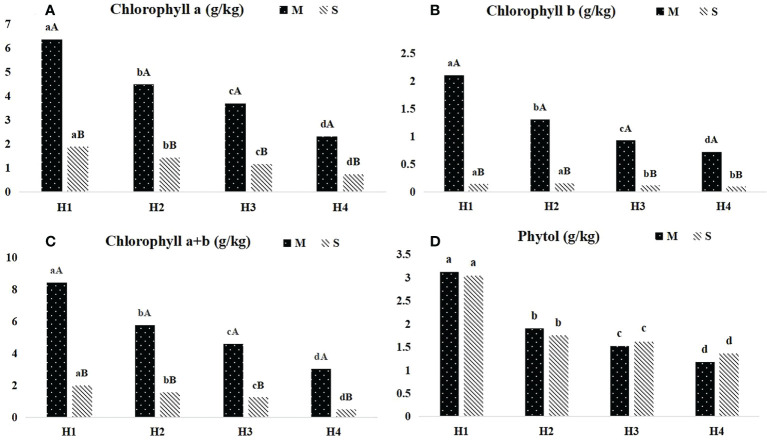
Dynamics of chlorophyll and phytol content in different height *Pennisetum sinese* and silage. The contents of chlorophyll a, chlorophyll b, chlorophyll a+b and phytol **(A–D)**. M: *Pennisetum sinese* material; S: *Pennisetum sinese* silage. Boxes with a different letters above the error bars are significantly different between different height **(a–d)** or material and silage **(A, B)** at *P* < 0.05.

The content of chlorophyll and phytol of *Pennisetum sinese* at different heights before and after ensiling were presented in **Figure 1**. The contents of chlorophyll a, chlorophyll b, and chlorophyll a+b significant decreased with the increase of plant height (*P* < 0.05), and the similar tendency also found in *Pennisetum sinese* silage (*P* < 0.05). Meanwhile, silage significantly reduced the chlorophyll a, chlorophyll b and chlorophyll a+b content (*P* < 0.05). Furthermore, there was no significant difference in phytol content before and after ensiling.

**Figure 2:**
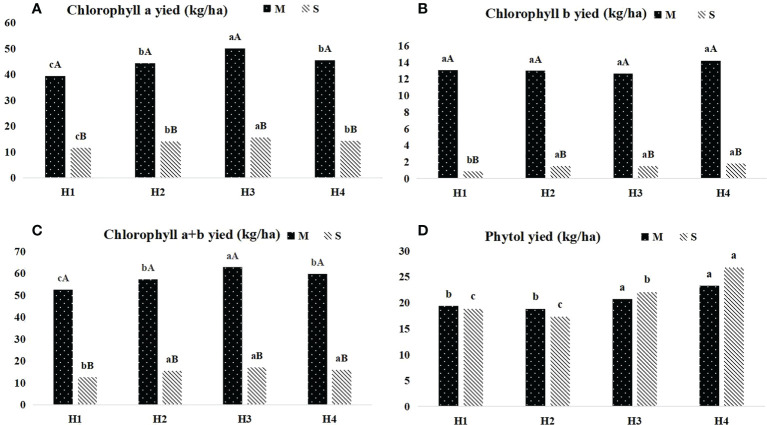
Dynamics of chlorophyll and phytol yield in different height *Pennisetum sinese* and silage. The yied of chlorophyll a, chlorophyll b, chlorophyll a+b and phytol **(A–D)** .M: *Pennisetum sinese* material; S: *Pennisetum sinese* silage. Boxes with a different letters above the error bars are significantly different between different height **(a–c)** or material and silage **(A, B)** at *P* < 0.05.

The dynamics of chlorophyll and phytol yield of *Pennisetum sinese* at different heights before and after ensiling were presented in **Figure 2**. The yield of chlorophyll a and chlorophyll a+b *Pennisetum sinese* before and after ensiling shown the inverted “V” trend, and H3 group had the highest yield (*P* < 0.05). Meanwhile, the highest chlorophyll b and phytol yield were in H4 group (*P* < 0.05). Furthermore, there was also no significant difference in phytol yield before and after ensiling.

### Silage fermentation quality of *Pennisetum sinese* in different heights

The dynamics of different height *Pennisetum sinese* silage fermentation characteristics list in **Table 2**. The plant height affect the fermentation characteristics of *Pennisetum sinese* silage, the highest lactic acid (*P* < 0.01), and lowest pH, acetic acid and propanoic acid (*P* < 0.05) were found in H3 group. The lowest and highest V-Score value were 55.52 in H1 group (*P* < 0.05) and 74.55 in H3 group (*P* < 0.05), respectively. *Pennisetum sinese* silage quality in H3 group was the best.

**Table 2:** Dynamics of different height *Pennisetum sinese* silage fermentation characteristics (DM).

	pH	Lactic acid (%)	Acetic acid (%)	Propanoic acid (%)	Butyrate (%)	NH_3_-N/TN (%)	V-Score
H1	3.91a	2.04b	8.26a	0.15a	0.41a	5.84	55.52c
H2	3.86b	1.93b	6.70b	0.21a	0.41a	5.44	56.32c
H3	3.62c	3.46a	0.61d	0.02b	0.26b	5.67	74.55a
H4	3.95a	2.07b	4.31c	0.12a	0.23b	5.94	69.72b
SEM	0.22	0.19	0.48	0.02	0.03	0.64	4.79
*P*-value	< 0.05	< 0.01	< 0.01	< 0.01	< 0.05	0.76	< 0.05

SEM, standard error of means. Means within the same column with different letters are significantly different (P < 0.05).

### Silage microbial communities of *Pennisetum sinese* in different heights

**Figure 3:**
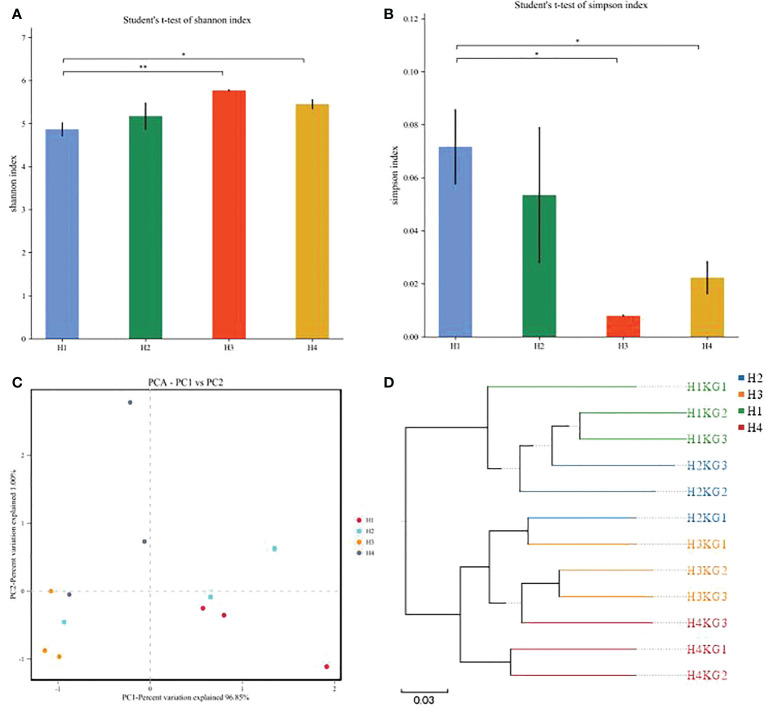
The α diversity and β diversity of different height *Pennisetum sinese* silage bacterial community. The Shannon and Simpson index **(A, B)** of the silage bacterial community, PCA and UPGMA analysis **(C, D)** of silage bacterial community. P-values are shown as *P < 0.05, **P < 0.01.

The silage microbial diversity of *Pennisetum sinese* was shown in **Figure 3**. The significant differences were observed in Shannon and Simpson indices between four groups (**Figures 3A, B**). The highest Shannon and lowest Simpson indices were in H3 group, which indicated H3 group had the highest microbial diversity. The PCA and cluster tree of the silage microbial community structures were conducted, and the clear separation and differences in bacterial community were found in all four groups (**Figures 3C, D**), which indicating that the microbial composition was changed by growth height, and microbial community structure was similar when the growth height was closer. Overall, above results demonstrated that growth height affects the microbial diversity and community structure of *Pennisetum sinese* silage.

**Figure 4:**
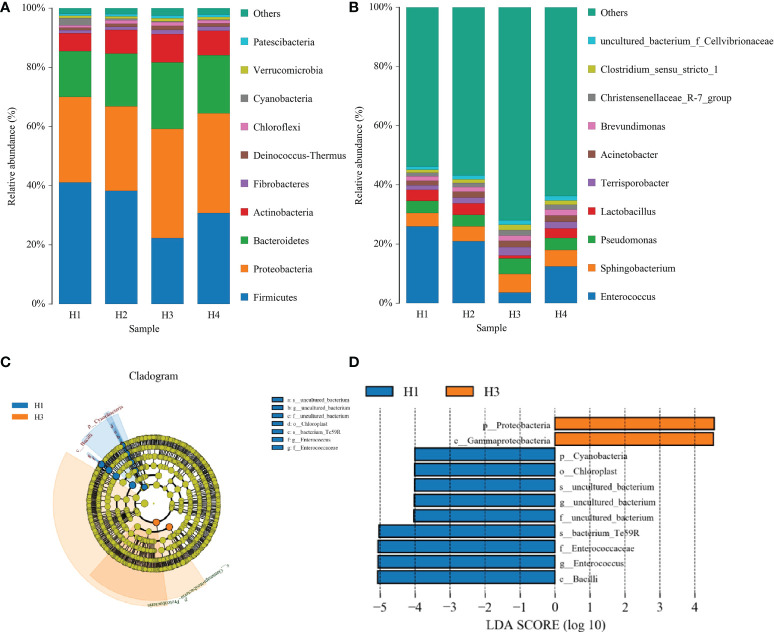
The composition (phylum, **A** and genus, **B**) and comparison using the LEfSe online tool **(C, D)** of the bacterial community of different height *Pennisetum sinese* silage.

**Figures 4A, B** shows the effects of growth height on the relative abundance of bacteria in the most dominant phylum and genus in *Pennisetum sinese* silage. The community was shifted along with different growth height: The abundances of *Proteobacteria*, *Bacteroidetes*, and *Actinobacteria* were increased, whereas the abundance of *Firmicutes* was decreased with rise of growth height. The H3 group was the highest abundance of *Proteobacteria*, *Bacteroidetes*, and *Actinobacteria*, and the lowest of *Firmicutes* (*P* < 0.05). Furthermore, the abundances of *Enterococcus* and *Lactobacillus* were decreased with rise of growth height, while the abundances of *Sphingobacterium* and *Pseudomonas* were increased. Meanwhile, the H3 group was the highest abundance of *Sphingobacterium* and *Pseudomonas* and the lowest of *Enterococcus* and *Lactobacillus* (*P* < 0.05).

The specific bacterial community of different growth height *Pennisetum sinese* silage was explored by the LDA effect size (LEfSe) method (LDA score > 4.0). **Figures 4C, D** shows that the different height of *Pennisetum sinese* used to produce the silage exerted a dramatic impact on the resultant microbial community. *Proteobacteria* and *Gammaproteobacteria* were the most abundant phylum and class in H3 group, respectively. In addition, *Cyanobacteria*, *Bacilli*, *Chloroplast*, *Enterococcaceae*, and *Enterococcus* were the most abundant phylum, class, order, family, and genus in H1 group, respectively. Moreover, these microorganisms could be used as biomarkers of the different height of *Pennisetum sinese* silage.

### Correlations between chlorophyll, phytol content and microbial communities of *Pennisetum sinese* silage

**Figure 5:**
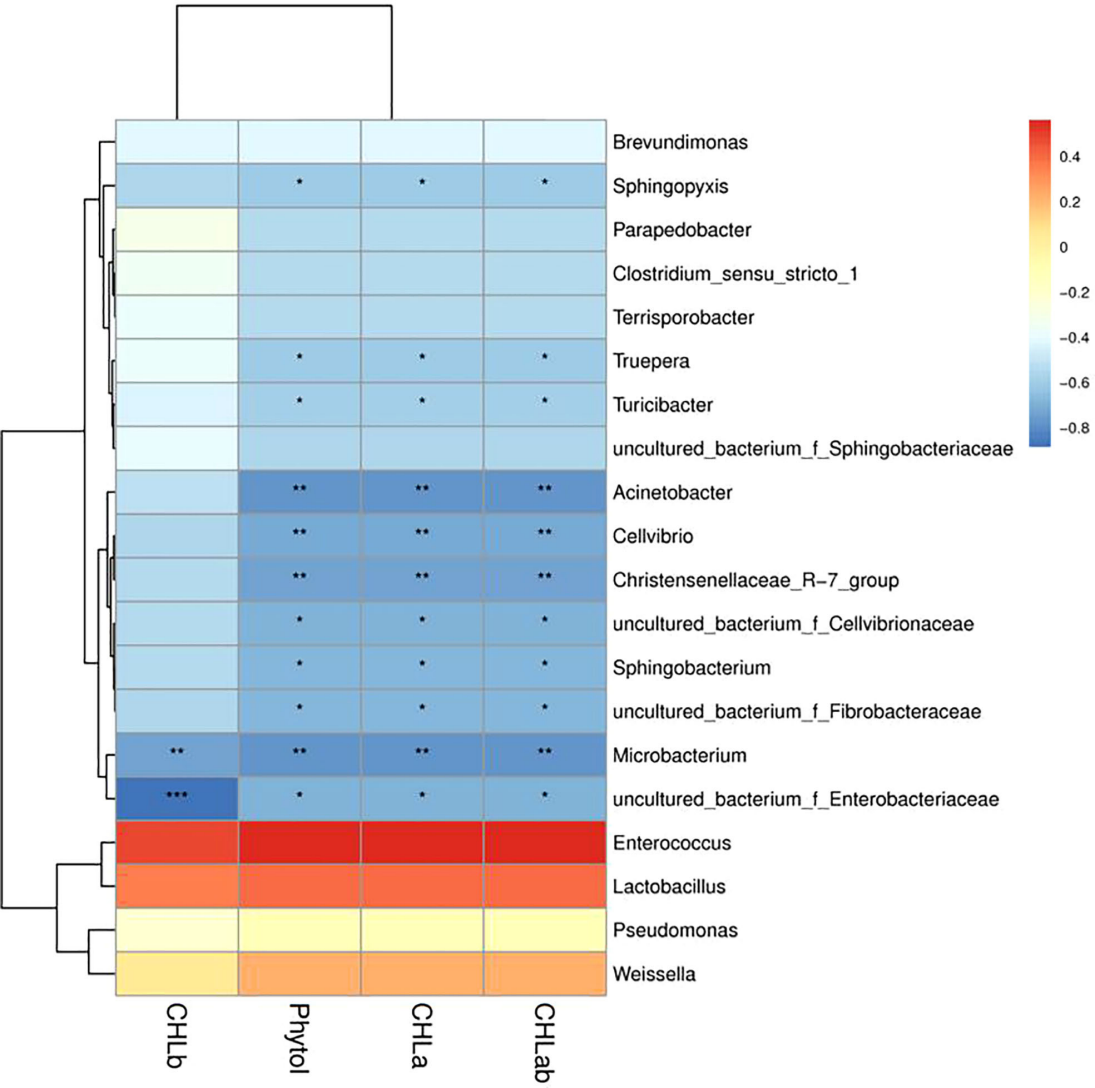
Correlations analysis between chlorophyll and phytol content and silage bacterial of *Pennisetum sinese.* Positive correlations are shown in red, and negative correlations are indicated in blue. Color intensity is proportional to the correlation values [r]. *P*-values are shown as *P < 0.05, **P < 0.01, ***P < 0.001.

The correlation between chlorophyll, phytol content, and microbial communities (the genus level) of *Pennisetum sinese* silage were assessed (**Figure 5**). The chlorophyll a, chlorophyll b, chlorophyll a+b, and phytol were no significant positively associated with *Enterococcus*, *Lactobacillus*, *Pseudomonas*, and *weissella*, whereas it was significant negatively associated with *Acinetobacter*, *Cellvibrio*, *Sphingobacterium*, *Christensenellaceae*_R-7_group, *Microbacterium*, *Sphingopyxis*, *Truepera*, *Turicibacter*, uncultured_bacterium_f_*Cellvibrionaceae*, uncultured_bacterium_f_*Enterobacteriaceae*, and uncultured_bacterium_f_*Fibrobacteraceae*. The result revealed that silage microbial maybe influence the degradation of chlorophyll and phytol during ensiling process.

## Discussion

In this study, the growth heights affected the chemical composition, chlorophyll, phytol, and biomass yield of *P. sinese*, and this result has been verified in several studies (King et al., 2013; Lv et al., 2017; Zi et al., 2017). The concentration of pigment decreased gradually with the increase of the growth height of *P. sinese*. On the contrary, DM yield increased with growing height. That result in pigment yield was higher in H3 or H4. In addition, the pigment content of *P. sinese* was reported for the first time in this study, the content were significantly higher than Italian ryegrass (Lv et al., 2017; Lv et al., 2020; Lv et al., 2021). Besides the cultivation conditions, the plants characteristics (variety or harvesting stage) also determine the differences in photosynthetic pigment content (Lv et al., 2017; Lv et al., 2021). As a kind of typical plant, the photosynthesis would be more effective under full sunlight, thus *P. sinese* produce the photosynthetic pigments more beneficial. However, Italian ryegrass is native to temperate zone with low photosynthetic efficiency and affected pigment synthesis. Furthermore, after ensiling the pigment in *P. sinese* markedly decrease, but not in phytol. Therefore, silage is an ideal way to preserve phytol compare with hay. Lv et al. (2017; 2020) reported the similar phenomenon in Italian ryegrass silage. Moreover, many studies have shown that forage at different growth stages has different silage qualities (Neylon and Kung, 2003; Lv et al., 2017). This could be explained by chemical differences at different growth stages. In this study, the best silage quality were found in H3 group ,which shown the highest V-Score value. Therefore, with comprehensive consideration of forage value, pigment yield, and silage quality, the optimum growth height of *P. sinese* was H3 (150 cm).

In this study, the PCA and cluster tree results showed separation and difference of microbial communities of *P. sinese* silage. The specific changes were reflected by the differences in bacterial composition at the genus level among different growth heights treatments silage. In general, *lactobacillus* is the dominant organism in well-preserved forage silage (Zi et al., 2021; Du et al., 2022). However, in the present study, *Enterococcus*, *Sphingobacterium*, and *Pseudomonas* were predominant, and *Enterococcus* has the highest abundance, that means the fermentation quality is not ideal enough. Then, we found an interesting phenomenon, the abundances of *Enterococcus* were reduce significantly, whereas *Sphingobacterium* and *Pseudomonas* were increased with rise of growth height, and then the fermentation quality was also improved. *Enterococcus* was aerobic fermentative Gram-positive coccus, which can utilize wide range of fermentable carbohydrates, mainly produced L(+) -lactic acid (Murray, 1990). It is a common microorganism in silage, especially at the beginning of ensiling, so it belongs to silage beneficial microorganisms at some extent (Bal et al., 2012; Wang et al., 2019). *Sphingobacterium* can consume carbohydrates and produce acid (Yabuuchi et al., 1983; Palleroni and Bradbury, 1993; Li et al., 2021). *Pseudomonas* is generally regarded as undesirable microorganisms for silage due to its possibility decreased protein content (Santos Silla, 1996; Dunière et al., 2013; Li et al., 2021). However, Ogunade et al. (2018) reported that these microorganisms were negatively correlated with pH, ammonium nitrogen, yeast, and mold and thought that they may contribute to silage fermentation. Moreover, in this study, *Sphingobacterium* and *Pseudomonas* have relative lower abundances, and which significantly increased in higher growth heights groups and improved the silage quality. Therefore, it is necessary to conduct in-depth studies on the characteristics and functions of these uncommon silage microorganisms.

In order to resolve the regulation mechanism of silage fermentation, some previous studies explored the correlation between silage microbial and fermentation characteristics, forage chemical composition or silage metabolites, and some key chemical components or metabolites were obtained, which could be used for precise control of silage fermentation quality (Xu et al., 2019; Wu et al., 2020; Zi et al., 2021; Li et al., 2021; Du et al., 2022). The variation of pigment and phytol content in silage were reported, which was consistent with this study, that was silage significantly reduced the pigment content but had no significant effect on phytol content (Lv et al., 2017; Lv et al., 2020). Spearman correlation analysis between silage microbial and pigment and phytol content was conducted; some specific undesirable microorganisms were significant negatively associated with pigment and phytol content. The results suggest that it is possible to increase pigment and phytol content by reducing the specific undesirable microorganisms. Nevertheless, studies have shown that phytol can be preserved well in silage, and the fermentation quality did not affect its content (Lv et al., 2017; Lv et al., 2020). However, the role of silage microorganisms in pigment degradation is also unclear. Usually, it is certain that the forage with good fermentation quality is more conducive to long-term preservation. Therefore, in order to reduce the pigment degradation and silage well storage, it is of great significance to control the specific undesirable microorganisms in silage.

## Conclusions

This study explored the dynamics of chlorophyll and phytol contents in different growth heights of *P. sinese* before and after silage and their relationship with silage microbial. The pigment content of *P. sinese* decreased with the increase of growth height and the difference of pigment content before and after silage was significant (except phytol). In addition, the pigment yield before and after silage at different growth heights were of significant differences, and yield of height H3 or H4 were obviously higher. Moreover, there were diverse fermentation quality and microbial community of *P. sinese* silage at different growth heights, and some undesirable microorganisms were negatively correlated with pigment content. Therefore, comprehensive consideration of pigment, phytol yield and silage quality, the optimum harvest growth height of *P. sinese* was 150 cm.

## Data Availability Statement

The datasets presented in this study can be found in online repositories. The names of the repository/repositories and accession number(s) can be found in the article/supplementary material.

## Author contributions

ML, RL, XZ, and HZ did the experimental design work. ML and RL conducted the experiments. ML, RL, XZ, and HZ analyzed the data. ML and XZ wrote and revised the manuscript. All authors read and approved the manuscript.

## Funding

This study was funded by the Natural Science Foundation of Hainan Province, China (322RC774), the Key Research and Development Program of Hainan Province (GHYF2022004), the Ministry of Agriculture and Rural Affairs of the People’s Republic of China (16220078) and the Central Public-Interest Scientific Institution Basal Research Fund for Chinese Academy of Tropical Agricultural Sciences (1630032022011).

## Conflict of interest

The authors declare that the research was conducted in the absence of any commercial or financial relationships that could be construed as a potential conflict of interest.

## Publisher’s note

All claims expressed in this article are solely those of the authors and do not necessarily represent those of their affiliated organizations, or those of the publisher, the editors and the reviewers. Any product that may be evaluated in this article, or claim that may be made by its manufacturer, is not guaranteed or endorsed by the publisher.
